# Implementing electronic clinical reminders for lipid management in patients with ischemic heart disease in the veterans health administration: QUERI Series

**DOI:** 10.1186/1748-5908-3-28

**Published:** 2008-05-29

**Authors:** Anne Sales, Christian Helfrich, P Michael Ho, Ashley Hedeen, Mary E Plomondon, Yu-Fang Li, Alison Connors, John S Rumsfeld

**Affiliations:** 1 University of Alberta, Edmonton, Alberta, Canada; 2 VA Puget Sound Health Care System, Seattle, Washington, USA; 3 VA Eastern Colorado Health Care System, Denver, Colorado, USA; 4 University of Denver Health Sciences Center, Denver, Colorado, USA

## Abstract

**Background:**

Ischemic heart disease (IHD) affects at least 150,000 veterans annually in the United States. Lowering serum cholesterol has been shown to reduce coronary events, cardiac death, and total mortality among high risk patients. Electronic clinical reminders available at the point of care delivery have been developed to improve lipid measurement and management in the Veterans Health Administration (VHA). Our objective was to report on a hospital-level intervention to implement and encourage use of the electronic clinical reminders.

**Methods:**

The implementation used a quasi-experimental design with a comparison group of hospitals. In the intervention hospitals (N = 3), we used a multi-faceted intervention to encourage use of the electronic clinical reminders. We evaluated the degree of reminder use and how patient-level outcomes varied at the intervention and comparison sites (N = 3), with and without adjusting for self-reported reminder use.

**Results:**

The national electronic clinical reminders were implemented in all of the intervention sites during the intervention period. A total of 5,438 patients with prior diagnosis of ischemic heart disease received care in the six hospitals (3 intervention and 3 comparison) throughout the 12-month intervention. The process evaluation showed variation in use of reminders at each site. Without controlling for provider self-report of use of the reminders, there appeared to be a significant improvement in lipid measurement in the intervention sites (OR 1.96, 95% CI 1.34, 2.88). Controlling for use of reminders, the amount of improvement in lipid measurement in the intervention sites was even greater (OR 2.35, CI 1.96, 2.81). Adjusting for reminder use demonstrated that only one of the intervention hospitals had a significant effect of the intervention. There was no significant change in management of hyperlipidemia associated with the intervention.

**Conclusion:**

There may be some benefit to focused effort to implement electronic clinical reminders, although reminders designed to improve relatively simple tasks, such as ordering tests, may be more beneficial than reminders designed to improve more complex tasks, such as initiating or titrating medications, because of the less complex nature of the task. There is value in monitoring the process, as well as outcome, of an implementation effort.

## Background

Ischemic heart disease (IHD) is one of the leading causes of death in the United States' veteran population. It affects at least 150,000 veterans annually and is the primary diagnosis in approximately one out of 17 admissions to Veterans Health Administration (VHA) hospitals [1,2] Numerous studies have demonstrated that lowering serum cholesterol levels, specifically low-density lipoprotein cholesterol (LDC-c), reduces coronary events, cardiac death, and total mortality, with benefits accruing particularly to patients with pre-existing heart disease [3-7] In 1997, the VHA adopted comprehensive guidelines which followed recommendations of national organizations for treating patients with IHD and called for lowering LDL-c to 100 mg/dL or less in patients with known IHD [8-10]. However, research has indicated that veterans receiving primary care in VHA may not have had their LDL-c measured or received treatment with lipid-lowering agents at optimal rates [11,12].

Clinical practice guidelines are known to be difficult to implement. Many studies have tested interventions to improve adherence to clinical practice guidelines for a variety of conditions and in a range of settings, but even after intervention, these studies find wide variation in guideline adherence and fail to find any specific interventions consistently associated with improved adherence [13-18]. Several meta-analyses have suggested the need for a systems approach combining multiple interventions and addressing contextual factors [15,19-23] – although even here doubts have emerged [24]. Among individual interventions, electronic reminders have been found to be modestly effective in increasing adherence to certain types of guidelines, including screening guidelines [25], and reminders may be more effective, on average, than other interventions [15,16].

Prior studies have found that reminders are not consistently used by clinicians when they are made available [26-30]. Few have provided details of efforts made to implement and assist clinicians in learning how to use reminders that are available.

In this article, we report results of an exploratory study of a multi-site, multi-faceted quality improvement intervention tailored to local contexts and designed to implement electronic clinical reminders in order to improve rates of LDL-c measurement and pharmacologic management among VA IHD patients. The study was initially planned as a first step in designing a randomized controlled trial to implement a complex intervention [31], and was exploratory in nature. Our original intent had been to follow this preliminary study with a larger, multi-site study in which we had planned to test the effectiveness of a complex, multi-level, multi-faceted intervention. In the planned intervention, we would have tested, in part, the effectiveness of implementing clinical reminders with and without the type of facilitation we describe in this paper. For several reasons, this larger study did not proceed.

This article is one in a Series of articles documenting implementation science frameworks and approaches developed by the U.S. Department of Veterans Affairs Quality Enhancement Research Initiative (QUERI). QUERI is briefly outlined in Table 1 and is described in more detail in previous publications [32,33]. The Series' introductory article [34] highlights aspects of QUERI that are related specifically to implementation science, and describes additional types of articles contained in the Series.

**Table 1:** The VA Quality Enhancement Research Initiative (QUERI)

The U.S. Department of Veterans Affairs' (VA) Quality Enhancement Research Initiative (QUERI) was launched in 1998. QUERI was designed to harness VA's health services research expertise and resources in an ongoing system-wide effort to improve the performance of the VA healthcare system and, thus, quality of care for veterans.

QUERI researchers collaborate with VA policy and practice leaders, clinicians, and operations staff to implement appropriate evidence-based practices into routine clinical care. They work within distinct disease- or condition-specific QUERI Centers and utilize a standard six-step process:
1) Identify high-risk/high-volume diseases or problems.
2) Identify best practices.
3) Define existing practice patterns and outcomes across the VA and current variation from best practices.
4) Identify and implement interventions to promote best practices.
5) Document that best practices improve outcomes.
6) Document that outcomes are associated with improved health-related quality of life.

Within Step 4, QUERI implementation efforts generally follow a sequence of four phases to enable the refinement and spread of effective and sustainable implementation programs across multiple VA medical centers and clinics. The phases include:
1) Single-site pilot,
2) Small-scale, multi-site implementation trial,
3) Large-scale, multi-region implementation trial, and
4) System-wide rollout.

Researchers employ additional QUERI frameworks and tools, as highlighted in this *Series*, to enhance achievement of each project's quality improvement and implementation science goals.

## Methods

### Study design

We conducted a quasi-experimental study using a hospital level intervention to implement electronic clinical reminders with the goal of improving hyperlipidemia management in VA IHD patients. Intervention hospitals included three VHA hospitals and their satellite clinics on the eastern side of the Rocky Mountain Network (Sites A, B, and C), one of 21 regional networks within VHA. Comparison hospitals, in which no efforts were made to implement or encourage the use of the national clinical reminders, were the three VHA hospitals on the western side of the Rocky Mountain Network (Sites D, E, and F). In both the intervention and comparison groups, one of the three hospitals is a large, urban, tertiary hospital (Sites B and F), while the other two are smaller, non-tertiary hospitals in relatively small towns (Sites A, C, D, and E). In the three intervention hospitals, the two smaller hospitals (A and C) each had two to three satellite clinics, while the large hospital had eight satellite clinics.

We did not randomize sites to either intervention or comparison arms because of geographic differences between the two halves of the regional network, feasibility due to travel and budget restrictions, and because of concerns about the integrity of referral networks in each half of the regional network. This latter concern was expressed by the regional leaders who gave approval to conduct the intervention. Regional leaders advised working within the existing structure of the network as we conducted the intervention. Our original intent was to use a lagged design, introducing the intervention to the comparison half of the region following completion of the intervention in the first half. Because of delays in developing and releasing the reminders, we were not able to complete implementation in the comparison sites before the conclusion of the study period.

Primary care providers, consisting of general internists, family practitioners, nurse practitioners, nurses, and/or physician assistants, were the targets of the intervention. However, as we note below in our description of the reminders, the reminders could be viewed by other providers, such as health technicians or pharmacists, within the care team. We did not include these other providers in our training or facilitation efforts.

### The intervention

The intervention consisted of an internally and externally facilitated implementation of national electronic lipid clinical reminders to promote guideline-recommended secondary prevention for IHD and began with a kickoff meeting attended by interdisciplinary teams of three to eight primary care providers from each of the intervention hospitals (Table 2). To ensure identification and participation of local opinion leaders in the kickoff meetings, team members were selected through an iterative process of surveys, contacts with hospital and regional leadership, and expressions of interest on the part of clinicians.

**Table 2:** Intervention and comparison facility descriptions

	**Intervention**			**Comparison**		
	**Site A**	**Site B**	**Site C**	**Site D**	**Site E**	**Site F**

**Site description**	Small non-tertiary facility in a relatively small city; frontier state	Large tertiary teaching center in a large metropolitan area with several smaller clinics in outlying areas	Very small non-tertiary facility in a small city in extremely remote area	Relatively large non-tertiary outpatient only facility with several smaller clinics in outlying areas	Small non-tertiary facility in a relatively small city; frontier area	Large tertiary teaching center in a large metropolitan area with several smaller clinics in outlying areas

**Number of patients with IHD during entire study period**	1883	4440	1001	4021	1399	5763
**Number of primary care providers**	14	60	9	22	11	83
**Proportion of PCPs who responded to survey**	80%	78%	96%	80%	70%	57%
**Proportion of PCPs who are MDs**	65%	53%	79%	62%	71%	33%
**Proportion of PCPs who are over 45 years old**	60%	58%	50%	46%	43%	36%
**Proportion male PCPs**	57%	46%	81%	28%	73%	43%
**Proportion PCPs stating they feel clinical reminders are useful**	45%	49%	50%	71%	64%	39%

**Commitment to intervention at baseline**						

**Size of team attending kick off meeting**	3 of 8	8 of 21	4 of 9	NA		
**Composition of team attending kick off meeting**	1 MD, 2 RNs	1 QM, 1 Admin, 6 NPs or PAs	2 MDs, 2 RNs			

PCP-Primary Care Provider

The kickoff meeting included talks by local and national experts in cardiology and lipid management. Teams from each hospital participated in small group sessions reviewing known barriers and facilitators to implementing new practices within their hospitals, and discussed specific barriers and concerns about their hospitals. Participants completed surveys designed to measure their perceptions of organizational readiness to change, and discussed the aggregate findings in the context of preparing system change. They were trained in the installation and use of reminders and were provided with the necessary support to enable them to champion the implementation of the national electronic clinical reminders in their facilities.

Following the kickoff meeting, bi-monthly conference calls with intermittent one-on-one phone and email contact were held between all participating intervention team members and the lead intervention teams based in Seattle and Denver. The Seattle team consisted of the principal investigator, a project director who had overall responsibility for project management and coordination, and a programmer/analyst. The Denver team consisted of the co-PI, a project manager who had primary responsibility for contact with the intervention sites, and a programmer/analyst. Through such contact, teams were able to give reports and discuss barriers encountered. Teams that had overcome some of the identified barriers offered solutions to others. The intervention period was from June 2002 (when the kickoff meeting was held) through September 2003.

### The reminders

The two VHA national lipid clinical reminders were released in May 2002 as an addition to the VHA Computerized Patient Record System (CPRS). CPRS is a fully electronic medical record system with computerized order entry, including laboratory tests, medication ordering, and consultation [35]. The first reminder is triggered by the absence of an LDL-c value within the past 15 months for patients with documented IHD in their medical record, either in the problem list or as an ICD-9 code in the discharge codes for each visit or admission. It consists of a dialog box that reminds the provider that LDL-c testing is due and briefly describes the evidence for taking action. Check boxes within the dialog box permit the provider to directly order the required lab test. VHA CPRS electronic clinical reminders do not "pop up" for clinician viewing. Instead, once triggered, they appear in a folder that is available through the face page of the patient's record when it is first opened by the clinician. A reminders tab is available whenever the patient record is open. We conducted the intervention in part because of the passive nature of VHA clinical reminders, believing that additional championship and training would be required to encourage providers to use the reminders.

This reminder can be completed by primary care providers or ancillary clinical personnel, including nurses. The second reminder is triggered by a current LDL-c of 130 mg/dL or greater. It consists of a dialog box with options for treatment including check boxes for direct ordering of medications (*e.g*., statins). In both cases, providers have the option of checking a box indicating that the diagnosis of IHD is inaccurate, or that they have chosen not to take recommended action based on clinical judgment.

The reminders were developed and released nationally by the VHA and were available to every VHA facility [26,35]. However, their use was not mandated by VHA Central Office. Decisions were made locally regarding whether to activate reminders for a hospital, clinic, or individual provider. Previous research has documented extensive variation across the VA as to whether or not reminders are activated [26]. While both the intervention and control hospitals had access to the national reminders, the intervention to implement the reminders occurred only in the intervention hospitals. The reminders were installed in the intervention hospitals within a month after the kickoff meeting, although there was considerable variation among the intervention sites in when the reminders were activated. In two of the comparison hospitals, the national reminders were activated at some point during the intervention period, even though no specific implementation efforts were undertaken. We do not have information about when the reminders were activated in these two comparison facilities.

### Patient population

Patients with a diagnosis of IHD who received care at the intervention or comparison hospitals during the observation period of September 2002 through June 2003 (*i.e*., they had at least one primary care visit during this period) were eligible for this study. Patients were identified as having IHD if they had an ICD-9-CM code of 410.xx (acute myocardial infarction), 411.xx (other acute and subacute forms of ischemic heart disease), 412.xx (old myocardial infarction), or 414.xx (other forms of ischemic heart disease) in the VA National Patient Care Databases (NPCD), and if they had been seen in primary care in a VHA hospital at least twice in the past three years. The algorithm for patient identification has been previously described by Sloan and colleagues [12].

Patient-level data, including age, gender, race/ethnicity, co-morbid conditions, self-reported income, lab values, and medication prescriptions were obtained from three sources. One was the VA regional Decision Support System (DSS), which contains laboratory and other clinical information for all patient encounters. The second data source was the VA Pharmacy Benefits Management (PBM) database, which contains detailed medication data on all VHA patients. The third was the NPCD, which contains records of all inpatient admissions and outpatient encounters. The same patient-level data were available for patients in both intervention and comparison hospitals. Patient age at baseline, gender, race/ethnicity, self-reported income, and number of co-morbid conditions were used to adjust the patient level outcomes. Race/ethnicity was coded as white/non-white, where patients for whom race/ethnicity was missing in administrative data (27%) were coded non-white. We repeated the analysis coding these patients as white, or missing, and found that it did not affect the results. The following diseases were coded as co-morbidities, and each scored one in the count of co-morbid conditions: diabetes, renal disease, chronic heart failure, depression, stroke, peripheral vascular disease, and substance use disorder. These conditions have been related to lipid measurement and treatment in our prior studies. Human subjects review and approval was obtained from the relevant institutional review boards.

### Study measures

We tracked participation in the intervention by the teams in each intervention hospital through conference calls, email messages, and other contacts with the intervention teams during the course of the intervention period. We compiled the data from the tracking system to report on barriers experienced by the intervention teams during the course of the intervention, and report these as specific events experienced in each hospital in a barriers section at the beginning of the results section.

We collected clinic-level data from each hospital detailing the number of clinical reminders due for patients, as well as the number of reminders satisfied (*i.e*., an action was taken which met the predetermined criteria for satisfying the reminder) on a weekly basis for the last half of the intervention period (May to September 2003). Reminder counts were tabulated only for the last half of the intervention period because data were only available for this period.

**Figure 1:**
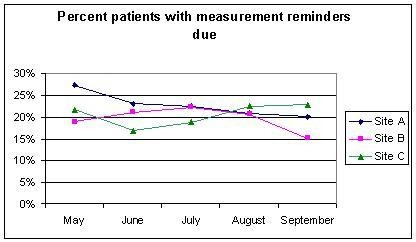
Percentage of patients at each of the three intervention sites with diagnosed IHD who had LDL-c measurement reminders due from May to September 2003.

**Figure 2:**
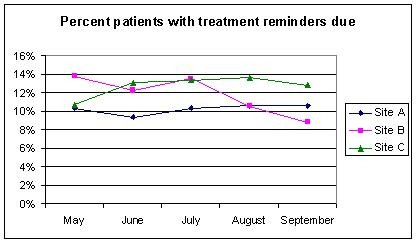
Percentage of patients at each of the three intervention sites who had a diagnosis of IHD and an elevated LDL-c measurement for whom treatment reminders were due from May to September 2003.

These data were available only in the intervention hospitals (Figures 1 and 2). In addition, we conducted a survey in June 2003, administered by email, of providers in both intervention and comparison hospitals, asking them about their use of and perceptions about electronic clinical reminders generally, and the IHD national clinical reminders in particular [Additional file 1]. Our information about use of reminders in the comparison hospitals and clinics comes from this survey.

The patient-level outcomes measured in this study included the changes in the proportion of IHD patients with current LDL-c measurement, and the proportion of patients with elevated LDL-c receiving lipid-lowering therapy to show the effect of the intervention on key process measures between June 2002 and September 2003 in both the intervention and comparison hospitals. In the first analysis, we did not control for the degree of reminder use, measured by the proportion of providers who report using IHD reminders frequently at each site (both intervention and comparison). In the second analysis, we controlled for the degree of reminder use.

### Analysis

For the process evaluation of the intervention, we assessed the degree to which hospitals varied in their patient and provider characteristics at baseline. We conducted a qualitative assessment of intervention team participants' views on their organizations readiness to adopt practice change. We monitored and graphed trends in lipid measurement and lipid levels among patients with IHD at the intervention sites throughout the majority of the intervention period. We reported frequency of reminder use at the intervention sites from the reports that are generated from the electronic reminders (reminder reports). We also assessed provider self-report data on their use of electronic clinical reminders, both the two IHD reminders and other locally developed reminders.

For the summative or outcome evaluation, we conducted bivariate analyses comparing the change in proportion of patients with current LDL-c measurement and the proportion of patients with elevated LDL-c who were receiving lipid-lowering medication between intervention and comparison hospitals and between the beginning and end of the intervention period, using analysis of variance and the F-statistic or tabulation with χ^2 ^for inference testing. We included only those IHD patients who were present in all time periods during the study period. We also conducted multivariable analysis using two multivariable logistic regression models: the first for positive change in current measurement of LDL-c (*i.e*., patients without current measurement at baseline who had current measurement at the end of the intervention), and the second for positive change in prescribing lipid lowering agents for patients with LDL-c greater than 130 mg/dL. We entered a variable indicating intervention site in the multivariable analysis, and we used a cluster correction to correct for clustering by hospital. Finally, we adjusted for provider self-report of reminder use, as this measured whether or not the reminder actually was used, rather than assuming use based on the allocation by hospital to intervention or not. All analyses were conducted using Stata version 9.0. Multivariable analyses were conducted using logistic regression with a binary dependent variable indicating improvement in measurement or lipid level, adjusted for clustering using Stata's "cluster" command. This command corrects the standard errors for the effect of autocorrelation due to hospital.

## Results

As shown in Table 2, there was significant variation between individual hospitals in the number of patients diagnosed with IHD, number of primary care providers, and other characteristics. This variability occurred across both the intervention and comparison hospital groups. The number of patients included in the analysis was 5438, with a slightly higher proportion in the comparison hospitals (Table 3). More patients were identified as having IHD over the study period, as shown in Table 2. However, only 5438 patients were present at the beginning and end of the study, forming the cohort we followed over time. The intervention hospitals had slightly younger, lower income, and lower proportion white and male patient populations than the comparison hospitals. They also differed in the proportion of patients with LDL-c measured at baseline, with the intervention hospitals having a slightly lower rate of measurement. There was no difference between the two groups in the proportion of patients with elevated LDL-c receiving lipid-lowering medications at baseline.

**Table 3:** Patient characteristics in both intervention and comparison VHA hospitals at baseline

**Patient characteristics**	**Intervention**	**Comparison**	**p-value**
	**N = 2372**	**N = 3066**	

**Mean age**	69.0 (s.d. 10.3)	70.7 (s.d. 9.6)	<0.001
**% male**	98.2	98.6	0.02
**% white**	66%	68%	0.02
**% income less than $20,000 per year**	62%	55%	<0.001
**Mean number of comorbidities**	0.99 (s.d. 1.01)	0.98 (s.d.0.98)	0.68
**% with current LDL-c at baseline**	91.2%	94.0%	<0.001
**% on lipid lowering agents at baseline among IHD patients with LDL-c > 130 mg/dL**	79.6%	80.2%	0.884

### Barriers to implementing the reminders

Barriers included: Use of a diagnosis code that was different from those used within the reminder logic to identify IHD patients (Site C only); inability to find reminder experts who could train clinicians in their use (Site A only); lack of IT support staff to install and turn on the reminders (primarily affecting Sites A and B); and an organizational merger that took priority over all other activities (Site B only). Of note, none of the sites indicated that time for clinicians to use the reminder was a significant barrier, although time burdens are consistently cited among the most frequent barriers to clinical reminders. The passive nature of the reminders may have been a factor in limiting the degree to which reminders presented a burden for providers.

Barrier resolution included changing coding practice at Site C (unknown amount of time to resolve); and scheduling a training session at Site A, with the reminder champion from Site C traveling to participate in the training to make it relevant for clinicians (three months to resolve). The two barriers encountered at Site B were not readily resolvable, although the local team and our implementation team worked diligently to ameliorate the situation. Overall, the local team member morale remained high and the teams remained engaged throughout the intervention period.

### Reminder use from reminder reports

The trend lines for measurement reminders due for each of the three intervention hospitals over the five-month period for which reminder reports were available is shown in Figure 1; the trends were mixed in the three hospitals. Lower levels of measurement and treatment reminders due are indicative of increased guideline compliance, thus the desired trend would be a downward slope. Site A displayed a trend towards a decrease in the proportion of IHD patients with measurement reminders due, while Site B increased slightly initially, then decreased, and Site C decreased initially and then increased. The trends for treatment reminders due were different and are shown in Figure 2; Site A started low and stayed relatively flat, while Site B initially decreased, then increased slightly, and finally decreased considerably, and Site C initially increased, stayed relatively flat, and decreased at the end of the intervention period. We present summary statistics from the reminder reports in Table 4.

**Table 4:** Process outcomes: Reminder reports (Intervention hospitals only)

	**Site A**	**Site B**	**Site C**
**Number of IHD patients identified by electronic lipid reminders at each site**	720	1404	475
**Proportion of IHD patients with electronic lipid reminders due for LDL measurement at beginning of study period**	27%	19%	21%
**Proportion of IHD patients with electronic lipid reminders due for LDL measurement at end of study period**	20%	15%	23%
**Proportion of patients with electronic lipid reminders due for lipid lowering treatment at beginning of study period**	10%	14%	11%
**Proportion of patients with electronic lipid reminders due for lipid lowering treatment at end of study period**	11%	8%	13%

### Reminder use from provider self-report (survey data)

The provider survey data from both the intervention and comparison hospitals showed considerable variation across the sites (Table 5). On average, comparison sites reported higher overall use of electronic reminders (98.2% versus 88.4%, p = 0.03), and their use was uniformly high (over 90% in all sites). Intervention hospitals reported higher use, on average, of general lipid reminders than comparison hospitals (38.8% versus 20.3%, p = 0.01), however, there was considerable variation within intervention and comparison groups.

**Table 5:** Process outcomes: Provider survey responses

				**Site**				**Site**			
							
	**Intervention hospitals overall**	**Comparison hospitals overall**	**p-value**	**A**	**B**	**C**	**p-value**	**D**	**E**	**F**	**p-value**
Proportion of primary care clinicians who report using any electronic reminders whether the national IHD reminders or not *(from provider survey)	88.4%	98.2%	0.04	64.3%	94.9%	93.7%	0.01	100%	100%	96.4%	0.59
Proportion reporting frequent use of IHD electronic reminders	38.4%	20.3%	0.01	30.0%	36.4%	50.0%	0.38	45.8%	21.4%	2.8%	<0.001
Proportion reporting that IHD electronic reminders are very useful	32.3%	16.2%	0.02	35.0%	29.1%	37.5%	0.77	37.5%	14.3%	2.8%	0.001
Proportion reporting that IHD electronic reminders increase awareness of lipid monitoring for IHD patients	30.3%	14.9%	0.02	30.0%	25.4%	41.7%	0.35	29.2%	21.4%	2.8%	0.008
Proportion reporting that electronic reminder screens provide appropriate treatment/action options	27.3%	14.9%	0.06	25.0%	23.6%	37.5%	0.48	37.5%	7.1%	2.8%	

*Comparison sites had access to electronic IHD reminders, but were not encouraged to turn them on, nor was the implementation intervention deployed at these sites.

There was also variation in attitudes expressed by providers, in the degree to which providers reported that the IHD reminders were useful, with intervention hospitals generally reporting that they were more useful (32.3% versus 16.2%, p = 0.02); that they increased awareness of the need for measurement and treatment of these patients (30.3% versus 14.9%, p = 0.02); and, to a lesser degree, recommended appropriate treatment or action options (28.7% versus 15.8%, p = 0.06). There was less than maximal contrast in reminder use between intervention and control hospitals.

### Summative outcomes: Change in measuring and managing lipids

As shown in Table 6, the results of the summative evaluation showed that the intervention hospitals performed better overall in improving LDL-c measurement than did the comparison hospitals, adjusting for patient characteristics (odds ratio 1.96, 95% CI 1.43–2.88). However, there was no significant difference between the intervention and comparison hospitals in their treatment of patients requiring lipid-lowering medications. The effect of the intervention on lipid measurement was stronger for LDL-c measurement when the amount of reminder use, as reported by providers, was included in the adjustment (OR 2.35, 95% CI 1.96–2.81). Notably, the odds ratios for the two smaller intervention hospitals became insignificant after adjusting for self-reported reminder use, while the odds ratio for the large intervention site remained significant (OR 1.77, 95% CI 1.11–2.82).

**Table 6:** Summative outcomes: The proportion of patients with current LDL-c measurements and patients prescribed lipid lowering medications from baseline to the end of intervention period

	**Intervention versus Comparison**	**Individual Intervention Sites**			**Individual Comparison Sites**		
	**Intervention**	**Site A**	**Site B**	**Site C**	**Site D**	**Site E**	**Site F**

Odds ratio for change from baseline to end of intervention without adjusting for degree of implementation (95% confidence intervals)*							

Effect on proportion of patients with current LDL-c from baseline to end of intervention period¶	**1.96 (1.34,2.88)**	**1.45 (1.38,1.52)**	**1.57 (1.44,1.72)**	**1.64 (1.53,1.75)**	**0.57 (0.50,0.66)**	**0.67 (0.65,0.68)**	Reference
Effect on proportion of patients on lipid-lowering medications from baseline to intervention period¶	0.92 (0.72,1.19)	**0.89 (0.87,0.91)**	**1.10 (1.01,1.20)**	**0.54 (0.53,0.56)**	**1.30 (1.07,1.57)**	**0.68 (0.66,0.71)**	Reference

Odds ratio for change from baseline to end of intervention adjusting for provider self-reported amount of use of IHD reminder (95% CI)							

Effect on proportion of patients with current LDL-c from baseline to end of intervention period	**2.35 (1.96,2.81)**	1.33 (0.93,1.89)	**1.77 (1.11,2.82)**	1.46 (0.77,2.76)	**0.34 (0.27,0.43)**	**0.58 (0.45,0.75)**	Reference
Effect on proportion of patients on lipid-lowering medications from baseline to intervention period	0.87 (0.67,1.13)	0.85 (0.71,1.03)	1.05 (0.82,1.35)	**0.46 (0.34,0.61)**	1.35 (0.97,1.90)	**0.66 (0.59,0.75)**	Reference

*Adjusted for patient baseline and facility characteristics in Table 2; odds ratios significant at p < 0.05 **bolded**

§Intra-class correlation for measurement change was 0.08, 95% CI 0.00 – 0.18

¶Intra-class correlation for treatment change was 0.02, 95% CI 0.00 – 0.05

The odds of treatment for patients requiring medications was somewhat lower for intervention sites after adjusting for reminder use, but remained statistically indistinguishable from comparison sites.

## Discussion

The primary aim of this study was to explore the implementation of electronic clinical reminders in order to improve rates of LDL-c measurement and pharmacologic management among patients with known ischemic heart disease in VHA. There is no literature to date on the use of a hospital-level intervention to improve the use of electronic clinical reminders. However, consistent with prior papers reporting the results of process evaluation of a reminder intervention [29], we found that there appears to be an association between how much providers report using reminders with change in the patient-level outcome measures only for the measurement reminder.

Providers at all six sites (intervention and control) reported using reminders, although not necessarily the two specific reminders that were the subject of this implementation effort. We note that use of reminders is self-reported, and may not fully reflect actual use; in particular, providers may over-report use of reminders when asked to self-report. At the intervention hospitals, the measurement reminder (prompting the clinician to order a test measuring LDL-c when no current measurement was available in the record) appears to have been effective in increasing the proportion of patients with current LDL-c measurements. However, the treatment reminder (prompting clinicians to begin a medication when a patient was not on a lipid-lowering medication and had elevated LDL-c) appears not to have been effective, even when we took into account self-reported use of the reminder [19,20,22].

Data from the reminder reports suggested that the reminder due rates were not very high in the intervention hospitals, ranging from 19 to 27% for measurement and 10 to 14% for treatment (Table 4). Despite these low rates overall, there was more room for improvement in the measurement outcome than in the treatment outcome, and the lower response to the intervention for the treatment outcome may be related to the relatively low rate of reminders due at the beginning of the period when reminder reports became available. It is important to note that we did not have reminder reports until the latter part of the intervention period, and it is possible that the effect of reminders may have been greater earlier in the intervention period.

It is also important to note that VHA clinical reminders are passive – they do not "pop up" on the screen, but are housed in a reminders folder in the electronic health record. This requires that clinicians make an active effort to view the reminders folder in order to respond to clinical reminders. In our view, this increases the need for interventions to make clinicians aware of the reminders and learn how to use them, and may make it more important that clinicians have a favorable attitude towards reminders.

There were considerable differences among the sites in their use of other electronic clinical reminders prior to our initiating the intervention described in this paper. The comparison sites had existing electronic reminders for lipids and, in general, had higher levels of lipid reminder use than the intervention hospitals. While we were not able to determine exactly when electronic reminder use began in the comparison sites, it is likely that these sites had been early adopters of electronic reminders, and had been using them for a period of years prior to the intervention. Several papers have described problems in the user interface with electronic clinical reminders, including those used in VA [26-28,30]. Our findings demonstrate that difficulties may persist even when specific facilitation attempts are made, through training and support, to improve reminder use. It is notable that attitudes towards reminders reported by providers were more positive overall in the intervention than the comparison hospitals, despite the lower reported use of reminders.

This study also highlights the importance of including a comparison group when conducting studies designed to evaluate quality improvement interventions. If this study had consisted only of a pre-and post-intervention assessment of the change in proportion of measurement and treatment reminders due we may erroneously attributed significant changes in performance measures to the reminders. Having comparison sites allowed us to acknowledge that prior use of reminders was a critical factor in whether reminders were adopted or not, and whether they were used or not.

Prior to this study, we had completed work in several VA sites that revealed substantial performance gaps in measuring LDL-c and in treating high LDL-c levels (greater than 130 mg/dL) among veterans with IHD [12,36,37]. However, there were considerable delays in the development and testing of the national reminders. By the time we engaged in this implementation effort, trends had been improving in lipid measurement and management for IHD patients system-wide. It may have been advantageous, therefore, to have reassessed the level of performance gaps within these institutions prior to implementing the intervention. Alternatively, once developed, quality improvement interventions need to be rapidly implemented so that temporal changes in performance do not occur between baseline measurement and intervention implementation.

There is considerable literature on the effectiveness of reminders, much of which is undermined by not adjusting for either organizational or hierarchical variables, or for the degree of reminder use [29,38,39]. In this study, we controlled for the clustering inherent in an organization-level intervention and, as much as possible, for the degree to which the use of reminders may have affected outcomes. Our findings are consistent with a number of studies that have reported on the effectiveness of reminders[18-20,22,24].

Finally, our findings underscore the importance of formative and process evaluation in implementation research: first to maintain fidelity to the original intention of the intervention, and second to understand the degree of uptake of the implementation [29]. Our process evaluation included tracking conference calls and email messages, including content of discussion of implementation barriers and their resolution; a survey of providers asking about their use of reminders; and use of an informatics tool, the reports generated by reminders.

### Strengths and limitations

The quasi-experimental design was an important strength of this study which allowed us to evaluate the effects of the reminders in the intervention sites adjusting for temporal trends. However, because allocation to the intervention group was non-random, there is a threat from unobserved confounders. Comparison sites were also non-optimal because they had existing electronic reminders for lipids, and temporal trends in lipid performance measures may have been different for facilities with electronic reminders versus those without. An ideal control group would have been a matched set of sites without a reminder system. In addition, we were able to obtain reminders due and satisfied (process measure) data only for part of the intervention period. However, a strength of this study is having these data at all. Also it should be noted that the response rates at each site were variable (Table 2), a factor we were not able to control. In addition, we lacked reminder report data early on in the intervention period, when there may have been greater use of the reminders. Finally, this study was conducted in a single healthcare system, VHA, which is known for its advanced informatics capacity, and may not be easily generalized to other settings.

## Conclusion

Although the data suggest that the implementation effort may have had some impact, the effect of the implementation effort reported in this study is modest. This finding is consistent with reports of implementation efforts focused at the organizational level. Our study generated some new insights into how clinicians respond to reminders that focus on different aspects of a clinical problem, namely detection or screening versus medication initiation or intensification. This study also demonstrates the importance of including contemporary controls when evaluating quality improvement interventions. We also report some substantial barriers to implementing reminders at a facility level, including a possible significant effect of prior culture and attitudes towards reminders. Our findings suggest that assessing these factors is likely to be an essential component to successful implementation of electronic clinical reminders, and finding methods of intervening if negative attitudes or an unsupportive culture are present. It may be very important to have enough resources to respond to these barriers as part of an implementation plan.

## Competing interests

The authors declare that they have no competing interests.

## Authors' contributions

AS was the principal investigator of the study reported in this article, and she designed it in collaboration with other authors, supervised data collection, conducted analysis, and took the lead in drafting the manuscript, CH contributed to writing the manuscript and conducted the associated literature review, PMH participated in design, data collection, and writing the manuscript, AH was the project coordinator and conducted data collection and managed the intervention and survey, MEP was responsible for data extraction from VA national databases and data analysis, YFL designed the tracking database used in the process evaluation and assisted with data analysis, AC completed and updated the literature review and participated in writing the manuscript, JSR was co-principal investigator and participated as a site lead, participated in the intervention, supervised data collection and data extraction, and participated in writing the manuscript. All authors read and approved the final manuscript.

## Supplementary Material

Additional file 1 VA Clinical Reminder Provider Feedback Survey. A survey given to providers in both intervention and comparison hospitals, asking them about their use of and perceptions about electronic clinical reminders generally, and the IHD national clinical reminders in particular. Click here for file
