# Supplementary material for: Implementing electronic clinical reminders for lipid management in patients with ischemic heart disease in the veterans health administration: QUERI Series
Source: Implement Sci. 2008 May 29;3:28. doi: 10.1186/1748-5908-3-28 (PMC2423370; doi:10.1186/1748-5908-3-28)
Supplement: Additional file 1 — VA Clinical Reminder Provider Feedback Survey. A survey given to providers in both intervention and comparison hospitals, asking them about their use of and perceptions about electronic clinical reminders generally, and the IHD national clinical reminders in particular. [file 1748-5908-3-28-S1.doc]

VA Clinical Reminder Provider Feedback Survey

The VA National Ischemic Heart Disease (IHD) QuERI Project is conducting a survey to evaluate the usability of the VA electronic clinical reminders available in CPRS. The survey findings will be used to improve clinical reminder design. Participation is voluntary and all responses are confidential. Your feedback is very important to the success of this evaluation.

Please answer the following questions and return the survey in the enclosed envelope.

Is CPRS available in your patient rooms for use during clinic visits?

( YES

( NO

When do you usually use CPRS? (check all that apply)

( Before seeing each patient

( During each patient visit

( After each patient leaves

( None of the above

Are any electronic clinical reminders being used at your facility?

( YES

( NO

Do you use electronic clinical reminders?

( YES ( Please continue to the next question.

( NO ( Please skip to question #11.

For approximately what percentage of your clinic patients do you use electronic clinical

reminders and/or the clinical reminder due list in CPRS?

( 0-25% ( 26-50% ( 51-75% ( 76-100%

6. When you use electronic clinical reminders or the clinical reminder due list, on average, how many reminders are due for each patient?

For a new clinic patient (check one):

( None ( 1-2 ( 3-5 ( 6-10 ( 11-15 ( >15

For a return patient (check one):

( None ( 1-2 ( 3-5 ( 6-10 ( 11-15 ( >15

7. Please indicate the extent to which you agree or disagree with each of the following

statements regarding VA electronic clinical reminders. Circle the number that best describes your views.

Strongly Neither Agree Strongly

Agree Agree nor Disagree Disagree Disagree

a) They are very useful.

1 2 3 4 5

b) There are too many to be completed in a

single visit.

1 2 3 4 5

c) They are an efficient tool for improving

patient care.

1 2 3 4 5

d) They are generally easy to use.

1 2 3 4 5

e) They are unnecessary.

1 2 3 4 5

f) They increase my awareness of clinical

practice guidelines that apply to my

patients.

1 2 3 4 5

g) They increase my awareness of tests and other interventions that are due.

1 2 3 4 5

h) They interfere with clinical decision

making.

1 2 3 4 5

i) They improve the quality of patient care.

1 2 3 4 5

j) I don’t have time to use them.

1 2 3 4 5

8. Have you ever used the VA-IHD LIPID PROFILE or VA-IHD ELEVATED LDL electronic

clinical reminders?

( YES ( Please continue to the next question.

( NO ( Please skip to question #10.

9. Please indicate the extent to which you agree or disagree with each of the following

statements regarding the VA-IHD LIPID PROFILE and VA-IHD ELEVATED LDL electronic

clinical reminders. Circle the number that best describes your views.

Strongly Neither Agree Strongly

Agree Agree nor Disagree Disagree Disagree

a) I frequently use these reminders for my

IHD patients.

1 2 3 4 5

b) They appropriately identify my patients

who have a diagnosis of IHD.

1 2 3 4 5

c) They are very useful.

1 2 3 4 5

d) They increase my awareness of lipid monitoring for my patients with IHD.

1 2 3 4 5

e) The reminder screens provide appropriate options for optimal lipid measurement and control.

1 2 3 4 5

10. Which staff members routinely use electronic clinical reminders at your facility? (check one)

( Only providers (MD, DO, PA, NP)

( Only non-providers (nurses, ancillary staff)

( Both providers and non-providers

11. Please provide the following information about yourself and your practice.

a) Gender: ( Male ( Female

b) Age (years): ( 20-29 ( 30-44 ( 45-64 ( >64

c) Professional title/position (please check all that apply):

Title: ( MD ( DO ( PA ( NP

Position: ( Staff/Attending ( Fellow ( Resident

( Other (please specify) _______________________________

______

d) Medical specialty/subspecialty _____________________________________

e) How long have you worked in the VA system (years)? _______________

f) On average, how many half-days per week do you provide direct patient care in a VA outpatient clinic? (check one)

( None (1-2 ( 3-4 ( 5-6 ( 7-8 ( 9-10

g) Approximately how many patients do you see during an average half-day in a VA

outpatient clinic? (check one)

( 1-2 ( 3-4 ( 5-6 ( 7-8 ( 9-10 ( 11-12 ( >12

12. Please write-in any comments or suggestions you have regarding the VA-IHD clinical reminders or other VA electronic clinical reminders.

____________________________________________________________________________

____________________________________________________________________________

____________________________________________________________________________

Please return the survey in the enclosed envelope to:

Ashley Hedeen, MD, Project Director

VA National IHD QuERI Coordinating Center

Puget Sound Health Care System (S-152)

1660 S. Columbian Way

Seattle, WA 98108
